# A new AAV tool for highly preferentially targeting hippocampal CA2

**DOI:** 10.1186/s13041-023-01038-6

**Published:** 2023-06-11

**Authors:** Siqi Peng, Wenzhen Gu, Wenxiu Zhu, Yan Zhuang, Xiuqi Yang, Yaochen Lv, Sibie Meng, Wei Xie, Moyi Li

**Affiliations:** 1 School of Life Science and Technology, The Key Laboratory of Developmental Genes and Human Disease, Southeast University, Nanjing, 210096 China; 2 Jiangsu Co-Innovation Center of Neuroregeneration, Nantong University, Nantong, 226001 China

**Keywords:** Hippocampus, Cornu ammonis 2, AAV tool, Neural tracing, Neural activity regulation

## Abstract

Mice hippocampus contains three prominent subregions, CA1, CA3 and DG and is well regarded as an essential multiple task processor for learning, memory and cognition based on tremendous studies on these three subregions. The narrow region sandwiched between CA1 and CA3 called CA2 has been neglected for a long time. But it raises great attentions recently since this region manifests the indispensable role in social memory. Its unique physical position connecting CA1 and CA3 suggests the potential novel functions besides social memory regulation. But the CA2 is too small to be accurately targeted. A flexible AAV tool capable of accurately and efficiently targeting this region is highly demanded. To fill this gap, we generate an AAV expressing Cre driven by the mini Map3k15 promoter, AAV/M1-Cre, which can be easily utilized to help tracing and manipulating CA2 pyramidal neurons. However, M1-Cre labeled a small percentage of M1^+^RGS14^−^ neurons that do not colocalize with any RGS14^+^/STEP^+^/PEP4^+^/Amigo2^+^ pyramidal neurons. They are proved to be the mixture of normal CA2 pyramidal neurons, CA3-like neurons in CA2-CA3 mixed border, some CA2 interneurons and rarely few CA1-like neurons, which are probably the ones projecting to the revealed CA2 downstream targets, VMH, STHY and PMV in WT mice injecting this AAV/M1-Cre virus but not in Amigo2-Cre mice. Though it is still challenging to get a pure CA2 tracking and manipulation system, this tool provides a new, more flexible and extended strategy for in-depth CA2 functional study in the future.

**Supplementary Information:**

The online version contains supplementary material available at 10.1186/s13041-023-01038-6.

## Introduction

As one of the most studied brain regions, hippocampus is well known for its crucial roles in learning, memory and recognition due to the decades of unstopped study enthusiasm in this field. The best-known function of hippocampus is to act as the key regulator of episodic memory and spatial navigation [1, 2]. These functions are elucidated based on the studies focusing on three prominent subregions: The cornu ammonis 1 (CA1), cornu ammonis 3 (CA3) and dentate gyrus (DG). CA1 especially dorsal CA1 is where place cells identified to represent an animal’s location for the spatial recognition [3]. DG and CA3 are thought to be involved in multiple functions such as information processing and social recognition [4]. Meanwhile, anterior/posterior or ventral/dorsal hippocampus obviously execute different tasks [5]. For instance, dorsal CA1 contains place cells but ventral CA1 contains engram cells key for social memory [6, 7].

However, hippocampus contains another region, a narrow region sandwiched between CA1 and CA3, called CA2. CA2 has several distinguished features comparing to CA1 and CA3. The pyramidal neurons in CA2 have simple spines as CA1 pyramidal neurons but they have larger soma body size than CA1. While the pyramidal neurons in CA3 have thorny excrescences (complex spines) on their dendrites and similar soma body size as CA2 pyramidal neurons [8–10]. CA2 pyramidal neurons could be easily visualized by CA2 markers (RGS14, Amigo2, PCP4, STEP, MAP3K15, CACNG5, etc.) [9, 10]. But the boundary between CA2 and CA3 is still hard to be defined and a recent report regard CA2/CA3 border as a mixed region containing both of CA2 and CA3 neurons [11, 12].

The function of CA2 keeps mysterious for a long time. Until recently a bunch of studies indicate that this region actually plays unique roles in social memory formation [13–15]. And the neurologists believe that CA2 can function far more beyond social memory regulation [16] due to its unique anatomical position. CA2 even is expected as a central region in the coding of spatial, temporal and episodic memory [17]. More novel functions of CA2 are waiting to be revealed in different neurological states and activities. And it is absolutely necessary to distinguish anterior CA2 function from its posterior part. The problem is that regular stereotaxic surgeries or injections are too hard to accurately target this small area.

To realize accurate targeting to CA2 regions, Hitti and Kohara et al. constructed two knock-in mouse lines respectively, Amigo2-Cre and Map3k15-Cre, specifically labeling CA2 pyramidal cells [13, 18]. But lack of non-animal tools specifically or highly preferentially targeting CA2 region still hinder the pace to explore this area. Developing a viral system specifically or highly preferentially targeting CA2 is absolutely necessary so that this area can be labeled and manipulated spatially, temporally and genetically. In addition, it is not easy to acquire and maintain CA2 knock-in mice with the low cost. And it is time-consuming to wait until Cre-protein expression after adulthood. Therefore, we construct a virus able to highly preferentially target CA2 with the mini Map3k15 promoter. It can efficiently realize accurate manipulation in CA2 area as CA2 specific-Cre mice and it is much more flexible. Moreover, it can be utilized for specific gene deletion in CA2 region combined with flox/flox mice of other genes of interest so that the function of specific genes in this area can be highly preferentially studied.

## Materials and methods

### Animals

6–8 week-old male C57BL/6 mice were kept group-housed in 4 per cage. Inbred mouse strain Tg (Amigo2-cre) 1Sieg (Amigo2-Cre) was acquired from the Jackson Laboratory and inbred in the Animal Core Facility of Nanjing Medical University (Nanjing, China) for experiments. The mice were housed under standard laboratory conditions with access to food and water ad libitum, stable temperature (22 ± 1 °C), and 12-h light–dark cycle (lights on at 07:00). All animal care and experimental procedures were followed by the Animal Experimental Ethical Guide of Southeast University and Animal Core Facility of Nanjing Medical University.

### Constructs

The promoter of specific CA2 expression gene was identified based on Allen Brain Atlas and Mouse Genome Informatics. The sequences of mitogen-activated protein kinase kinase kinase 15 (Map3k15, NM_001163085.3, NC_000086.7) and regulator of G protein signaling 14 (RGS14, NM_001360714.1, NM_016758.3) were downloaded from the NCBI. Map3k15 and RGS14 promoter were amplified using mouse DNA as a template. Activity of promoters was detected by OIBO company (Shanghai, China).

And the following primers are used for the amplifications: 5′-gctggaactagggaaggatttc-3′ (Map3k15-2.3 kb promoter-F) & 5’-atcgtagaaggcatcgagcac-3’ (Map3k15-2.3kb promoter-R);

5′-ggaggaagtcagggagggaggaaa-3′ (Map3k15-0.5 k promoter-F3) & 5’-gcggggctggcggcttcgaa-3’ (Map3k15-0.5k promoter-R3);

5′-tgctccctatctctctgctttctg-3′ (RGS14-1.4 k promoter-F) & 5’-tctgccacaggaagtgtctcta-3’ (RGS14-1.4k promoter-R)

.

### Viruses and drugs

The specific CA2 promoter construction plasmids (rAAV-M1-NLS-CRE-WPRE-pA, M1 can be replaced by the M2 or RGS14 promoter) were packaged into Adeno-associated viruses (AAV)-M1-CRE (Map3k15-0.5 kb), AAV-M2-CRE (Map3k15-2.3 kb), and AAV-RGS14-CRE (RGS14-1.4 kb) by OBIO (Shanghai, China). AAV-CMV-EGFP (AOV022-1 AAV2/9), AAV-hSyn-DIO-mcherry (H4828 AAV2/9 CK1214 AAV2/9) were purchased from OBIO (Shanghai, China). AAV-Ef1a-DIO-EGFP (PT-0795 AAV2/5, PT-0012 AAV2/9), AAV-hSyn-CRE (PT0136, AAV2/9), AAV-Ef1a-DIO-hM4Di-mcherry (PT-0043 AAV2/9) were purchased from VTA (Wuhan, China). Clozapine-N-oxide (CNO, A3317) was purchased from APEXbio (TX, USA).

### Viral injections

P30 mice were anesthetized with isoflurane (2–5%) (RWD life Science Co, Shanghai, China) and had their scalp immobilized in a stereotaxic apparatus (RWD). After a precise craniotomy, the AAV was loaded in a glass pipette pulled by a heater, which was slowly targeted for injection into the dorsal hippocampus CA2 (left: A/P: − 1.55 mm, D/V: − 1.70 mm, M/L: − 1.70 mm; right: A/P: − 1.55 mm, D/V: − 1.72 mm, M/L: 1.63 mm). AAV-M1-CRE (2.99 × 10^12^ vg/ml) and AAV-hSyn-CRE (1.09 × 10^12^ vg/ml) were injected with equal amounts of viral particles by controlling the injection volume, and the volume ratio of AAV-CRE and AAV-DIO was ensured to be 2:3. A syringe pump (KD Scientific, Holliston, USA) was used to inject 100 nl of AAV into the dorsal CA2 region bilaterally at 10 nl/min. The needle was gradually withdrawn 20 min after injection. To prevent hypothermia after anesthesia, the mice were positioned on a heating pad through the procedure until fully awake. The injected mice were monitored daily for recovery status, body weight and infection.

### Behavioral test

Only 6–8 weeks old male C57BL/6 mice were used in behavioral test. For 3-chamber social test, two stranger mice were housed individually in their cages. The mice placed in the engagement area were age-matched, unfamiliar, and of the same genetic background and gender. To familiarize themselves with being held, the strange mice were acclimated 2 days in advance, 45 min a day, which was to avoid anxiety-induced odor and sounds from unfamiliar mice affecting the subjects’ activities. CNO was intraperitoneally injected at 0.5 mg/kg 20 min before the behavioral test.

### Open field test

The open field apparatus is a blue Plexiglas uncapped box (50 cm wide × 50 cm long × 50 cm high), which consists of two parts, the center (29.7 cm diameter in the center of the box) and the periphery. Mice were allowed to move freely for 10 min in the box. The movement of mice was recorded and analyzed with a webcam and Ethovision XT software (Noduls, Wageningen, Netherlands).

### Social memory test

The three-chamber behavior apparatus (60 cm long × 40 cm wide × 22 cm high) containing three compartments with equal size, which were separated by two walls with small doors (5 cm wide × 8 cm high). 20 min after intraperitoneal injection of CNO or vehicles, mice were placed in the device and allowed to explore freely for 10 min. The left and right chambers were placed with empty inverted uncapped cages before the experiment. Stranger mice (stranger 1, S1) were placed in the inverted cages in the chamber where the subject mice were not habitually explored. During the social novelty stage, the time that the test mice spent sniffing the novel mice and the empty cage in the other chamber was compared. After 10 min, the test mice were isolated in the middle chamber for 10 min, and at the same time a second novel stranger mouse (stranger 2, S2) was carefully put into the inverted cage that was originally empty in the previous stage. The subject mice were released from the middle chamber to explore the two compartments, and social preference was determined by comparing the time the mice took to sniff S2 and S1 in the last 10 min. Before introducing new subjects into the apparatus, the chambers and inverted cages were thoroughly cleaned with 25% ethanol. Behavior was monitored and scored for social novelty and memory which were represented by the anogenital and nose-to-nose sniffing time of the subject mice. Sniffing time was calculated by a trained observer, who was unaware of genotype and treatment of the mouse. The formulas, social difference score = Time_stranger1_-Time_empty_ (stage 1), and social difference score = Time_stranger2_-Time_stranger1_ (stage 2) were used to define the social novelty and social memory of the subject mice.

### Immunohistochemistry

After appropriate anesthesia with the CO_2_ bullets, animals were perfused intracardially with ice-cold PBS followed by 4% ice-cold PFA/PBS. Mouse Brains were fixed in 4% PFA overnight and then transferred into 30% sucrose for dehydration. After being embedded in Tissue-Tek^®^ O.C.T., the brains were snap-frozen in liquid nitrogen and stored at − 80 °C. The brains were sectioned into the slices with the thickness of 30 µm using a Leica cryostat (Leica CM1950, Wetzlar, Germany).

Brain serial sections were attached to glass slides coated with poly-L-lysine (Cat# P8920, Sigma, Germany), and then washed for 15 min with PBS. Heated-antigen retrieval was performed for staining. In general, water-bathed sections were incubated at 96 °C for 5 min in 50 mM sodium citrate pH 8.5 (P0083, Beyotime, Shanghai, China). After being washed from the incubator for 5 min, the sections were permeabilized in 0.3% Triton X-100 in PBS (PBT) for 30 min. Subsequently, the sections were blocked at room temperature for 2 h with 10% Fetal Bovine serum (FBS) in 0.3% PBT (10% FBST) before overnight incubation with primary antibodies at 4 °C diluted with 10% FBST (1:500). The next day, the sections were washed 7 times for 35 min in 0.3% PBT and stained with secondary antibody (1:500) in 10% FBST for 1 h, followed by 5 times washing in 0.3% PBT for another 25 min. After the sections were stained with DAPI (C1002, Beyotime), the slices were mounted. Images were captured under a Zeiss confocal microscope LSM 700 or LSM 900 (Zeiss, Oberkochen, Germany). The whole coronal brain image was taken by Palm Microbeam (Zeiss).

**Fig. 1:**
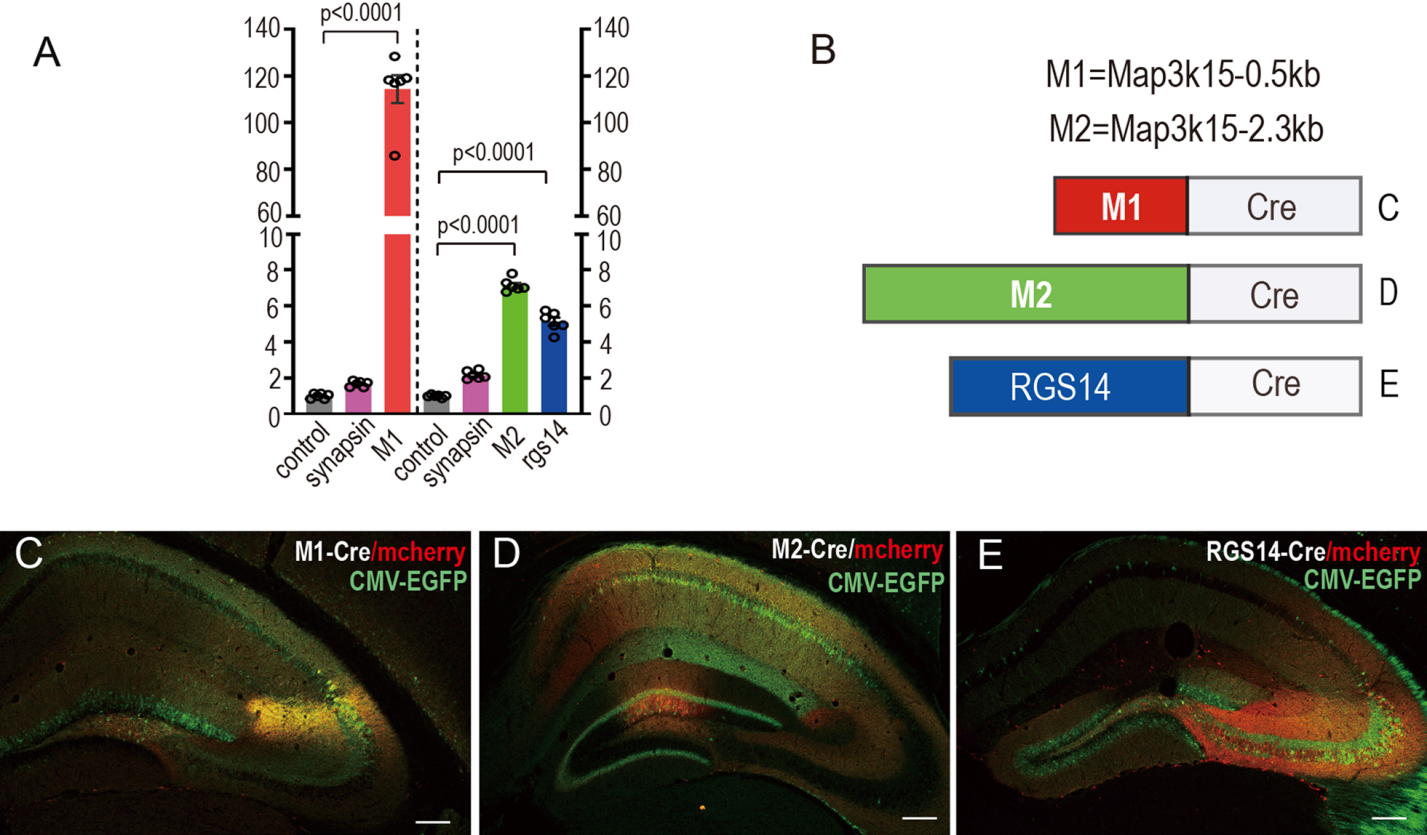
Comparison of the reporter expression driven by different CA2-specific promoters. **A** Comparison of dual-luciferase-expression efficiency driven by the long (2.3 kb) and short (0.5 kb) fragments of *map3k15* promoter and *rgs14* promoter (n = 6 mice). The graph showing the M1 (*map3k15, 0.5 kb*) promoter efficiency was 114 times higher than empty (p < 0.0001), while the M2 (*map3k15, 2.3 kb*) promoter was 7.125 times more efficient than the empty vector (p < 0.0001) and the *rgs14* promoter efficiency was 5.114 times higher than empty (p < 0.0001). Ordinary one-way ANOVA, post-hoc test Bonferroni’s test, each plasmids transfected 6 replicate wells, each column was the average value of firefly luciferase data divided by renialla luciferase after normalizing the pgl4.1 empty value. **B** Schematics of viral constructs containing different promoters driving Cre expression. **C** The image of dCA2 co-injected with AAV9/M1-Cre (0.5 kb), AAV9/hSyn-DIO-mcherry (red) and AAV9/CMV-EGFP (green) (n = 6 mice) showing the area of EGFP expression was larger than that of M1-mcherry positive cells (red), and their areas both were significantly larger than CA2 region. **D** The image of dCA2 injected with AAV9/M2-Cre, AAV9/hSyn-DIO-mcherry (red) and AAV9/CMV-EGFP (green) (n = 5 mice) showing the CMV-EGFP position is basically in CA1, and the number of M2-mcherry positive cells was relatively normal. But M2-mcherry cells were mainly located in DG and CA1 instead of CA2. **E** The image of dCA2 co-injected with AAV9/RGS14-Cre, AAV9/hSyn-DIO-mcherry (red) and AAV9/CMV-EGFP (green) (n = 4 mice) showing the area of EGFP expression was larger than that of red RGS14-mcherry positive cells, and their areas both were significantly larger than CA2 region. The RGS14-mcherry expression tends to diffuse outside from CA2 to CA3, and CMV-EGFP expression more tend to diffuse to DG. Scale bars: 200 μm (**C–E**)

**Fig. 2:**
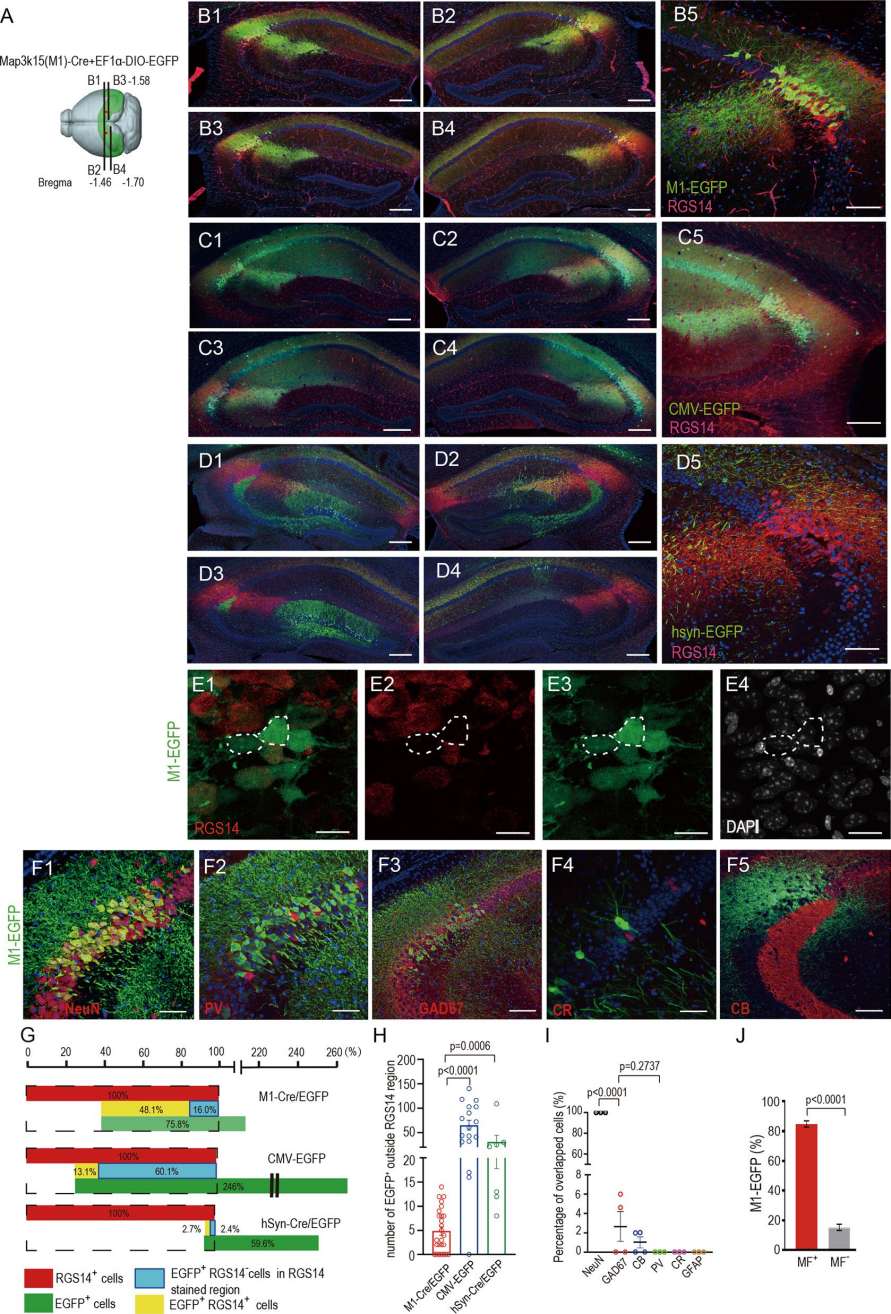
Comparison of EF1α-DIO-EGFP expression pattern driven by M1-Cre and by the non-CA2-specific Cre virus in dorsal CA2 of WT mouse and neuron type identification in the infected CA2. **A** Schematics of stereotactic virus injection at dorsal CA2 of WT mouse. **B1–B4** Representative confocal serial sections of hippocampus infected with M1-EGFP (green) and stained with RGS14 (red) (n = 6 mice). **B5** Magnified image of M1-EGFP (green) collocated with RGS14 (red) in CA2 region (n = 9 mice). **C1–C4** Representative confocal serial sections of hippocampus infected with CMV-EGFP (green) and stained with RGS14 (red) (n = 5 mice). **C5** Magnified image of CMV-EGFP (green) and RGS14 (red) (n = 9 mice). Image showing the expression range of CMV-EGFP exceeds the CA2 region, there were some EGFP and RGS14 overlap. **D1–D4** Representative confocal serial sections of hippocampus infected with AAV9/hSyn-Cre & AAV9/EF1α-DIO-EGFP (green) in dCA2 and stained with RGS14 (red) (n = 4 mice). **D5** Magnified image of hSyn driven EGFP (green) and RGS14 (red). Image showing no hSyn-EGFP cells and RGS14 overlapping, and EGFP expressed within CA2. **E1-E4** High magnification confocal images of CA2b displaying M1-EGFP unstained with RGS14 (dotted circles) and overlapped with RGS14. **F1** confocal image of dCA2 infected with M1-EGFP, showing M1-EGFP (green) and NeuN (red) colocalization (n = 3 mice). **F2** image showing no Parvalbumin (red) and M1-EGFP (green) colocalization (n = 3 mice). **F3** image showing no GAD67 (red) and M1-EGFP (green) colocalization in pyramidal cell layer (Py) but a little overlap in Oriens layer (Or) (n = 4 mice). **F4** image showing no M1-EGFP (green) and Calretinin (red) colocalization (n = 4 mice). **F5** image showing dCA2 infected with M1-EGFP stained with Calbindin (red). **G** Proportion of different cells in the hippocampus infected with M1-EGFP (AAV9/M1-Cre & AAV9/EF1α-DIO-EGFP or AAV5) (n = 6 mice) or CMV-EGFP (n = 4 mice) or AAV9/hSyn-Cre & AAV9/EF1α-DIO-EGFP (hSyn-EGFP) (n = 3 mice) on confocal coronal section. Black dash lines represent RGS14 stained CA2 region (Red bars). RGS14^+^ cells in RGS14 stained region were defined as total CA2 cells and is normalized as 100%. Green bars: the proportion of infected EGFP^+^ cells compared to RGS14^+^ cells; Yellow bars: the proportion of EGFP^+^RGS14^+^ cells to the total CA2 cells, Blue bars: the proportion of EGFP^+^RGS14^−^ cells to the total CA2 cells within RGS14 stained region. All means were calculated from no less than 3 slices for each biological sample. **H** Scatter chart displaying the number of EGFP^+^ neuron outside-RGS14-region in each treatment. Data are presented as mean ± SEM. **I** Quantitative analysis showing the percentage of M1-EGFP cells stained with various markers in (**F1–F4**). All means calculated from no less than 5 brain slices of each biological sample. **J** Quantitative analysis of M1-EGFP cells receiving Calbindin-positive mossy fibers (MF^+^) and not receiving mossy fibers (MF^–^) in **F5**. All sections above were stained with DAPI (blue). Scale bars: 100 μm (**B5, D5, F3, F5**), 200 μm (**B1–B4, C1–C4, D1–D4, C5**), 20 μm (**E1–E4**), 50 μm (**F1, F2, F4**)

**Fig. 3:**
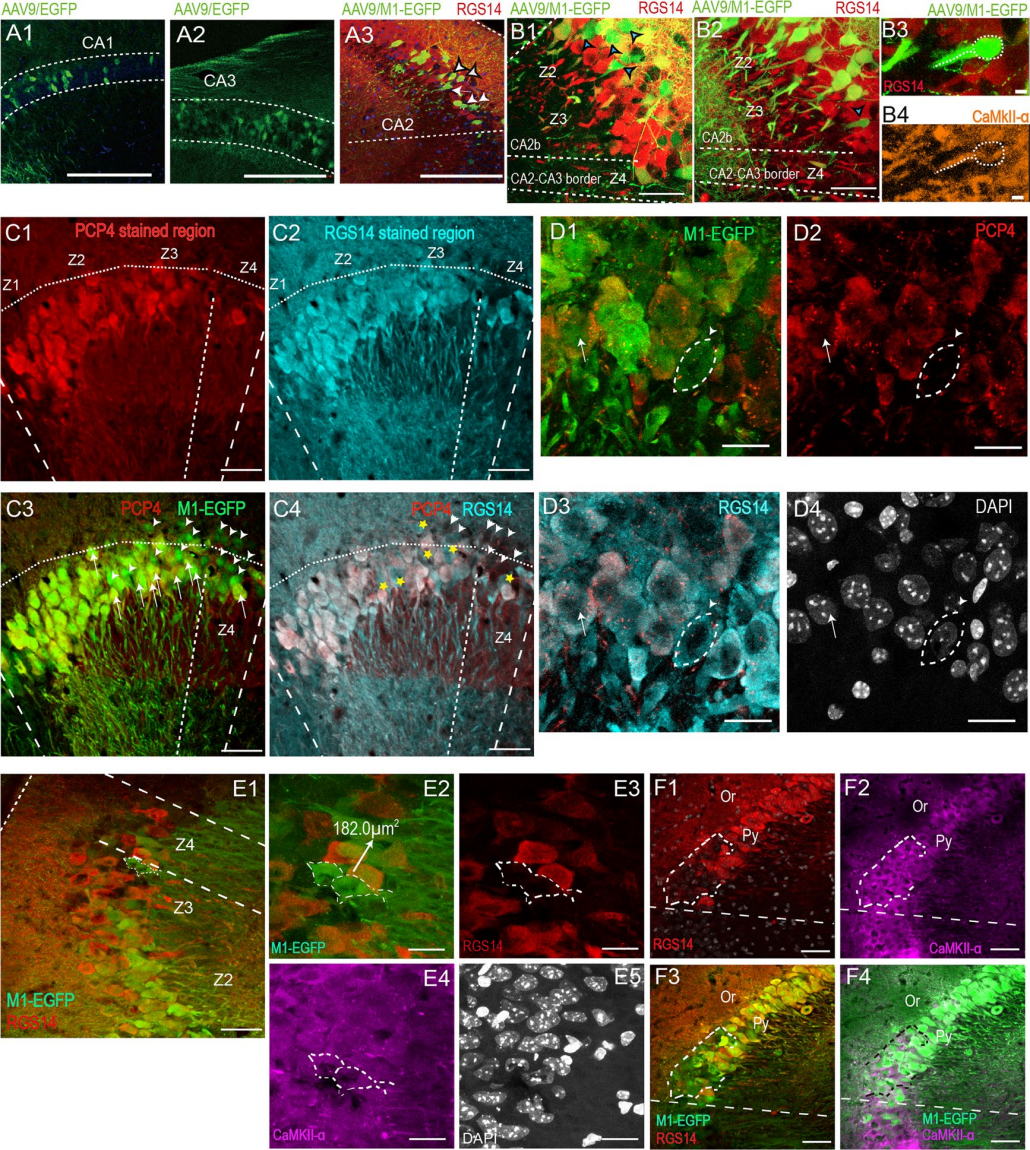
Molecular and cellular features of M1^+^RGS14^−^ neuron in CA2 Py layer. **A1–A2** Images of CA1 pyramidal neurons (**A1**) and CA3 pyramidal neurons (**A2**) infected with AAV9/EGFP. Dotted lines in A1 and A2 enclosed stratum pyramidale. **A3** Image of CA2 RGS14 positive and negative neurons infected with AAV9/M1-EGFP. Dotted lines enclosed RGS14 stained CA2 region (red). White arrows show M1^+^RGS14^−^ (M1 for Map3k15) neuron, white arrowheads with black edge show M1^+^RGS14^+^ neurons. **B1-B4** Images of CA2b pyramidal neurons infected with AAV9/M1-EGFP, (**B1, B2** the blue arrowheads with black edge indicate RGS14-failed expressed M1^+^ neurons). **B3 B4** High magnification images shows CA2b M1^+^RGS14^−^ overlapped with CaMKII-α in **B2** (the blue arrowhead with black edge). **C1–C4** IHC images showing M1-EGFP expressed neurons stained with PCP4 (**C1**) and RGS14 (**C2**). Superposed image of M1-EGFP and PCP4 (**C3**). Superposed image of PCP4 and RGS14 (**C4**). Dotted lines enclose CA2. White symbol in **C3** show M1^+^PCP4^−^ neurons (green) (arrowheads) and M1^+^PCP4^+^ neurons (yellow) (arrows). The dotted lines on Or divide the four zones of CA2. Yellow five-pointed stars in **C4** show RGS14^+^PCP4^−^. The standard for distinguishing Z1–Z4 is that Z1, Z2 each occupy 20% of the RGS14 staining area, Z3, Z4 each occupy 30%. Z1, Z2 occupy a small amount of space outside the cropped area. High magnification IHC images showing M1-EGFP expressed neurons stained with RGS14 (**D3**) and failed stained with PCP4 (arrow head), and arrow displays M1-EGFP stained with PCP4 (**D1, D2**). **E1** IHC image showing M1^+^RGS14^−^ neurons located in RGS14 stained region. Dotted lines enclosed RGS14 stained CA2 region (red). **E2–E5** Magnified images of the delineating area in F showing overlay between CaMKII-α (magenta) and M1^+^RGS14^−^. Dotted circles show M1^+^RGS14^−^ neurons. **F1–F4** IHC images showing M1-EGFP stained with RGS14 (**F1**) and CaMKII-α (**F2**). Superposed image of M1-EGFP and RGS14 (**F3**). Merged image of M1-EGFP and CaMKII-α (**F4**). Dotted circles indicate RGS14^−^EGFP^+^ neurons in **F3** and overlay with stained CaMKII-α in **F4**. Scale bars: 200 μm (**A1–A3**), 50 μm (**B1, B2, C1–C4, E1, F1–F4**), 20 μm (**E1–E4, D1–D4**), 2 μm (B3, B4)

**Fig. 4:**
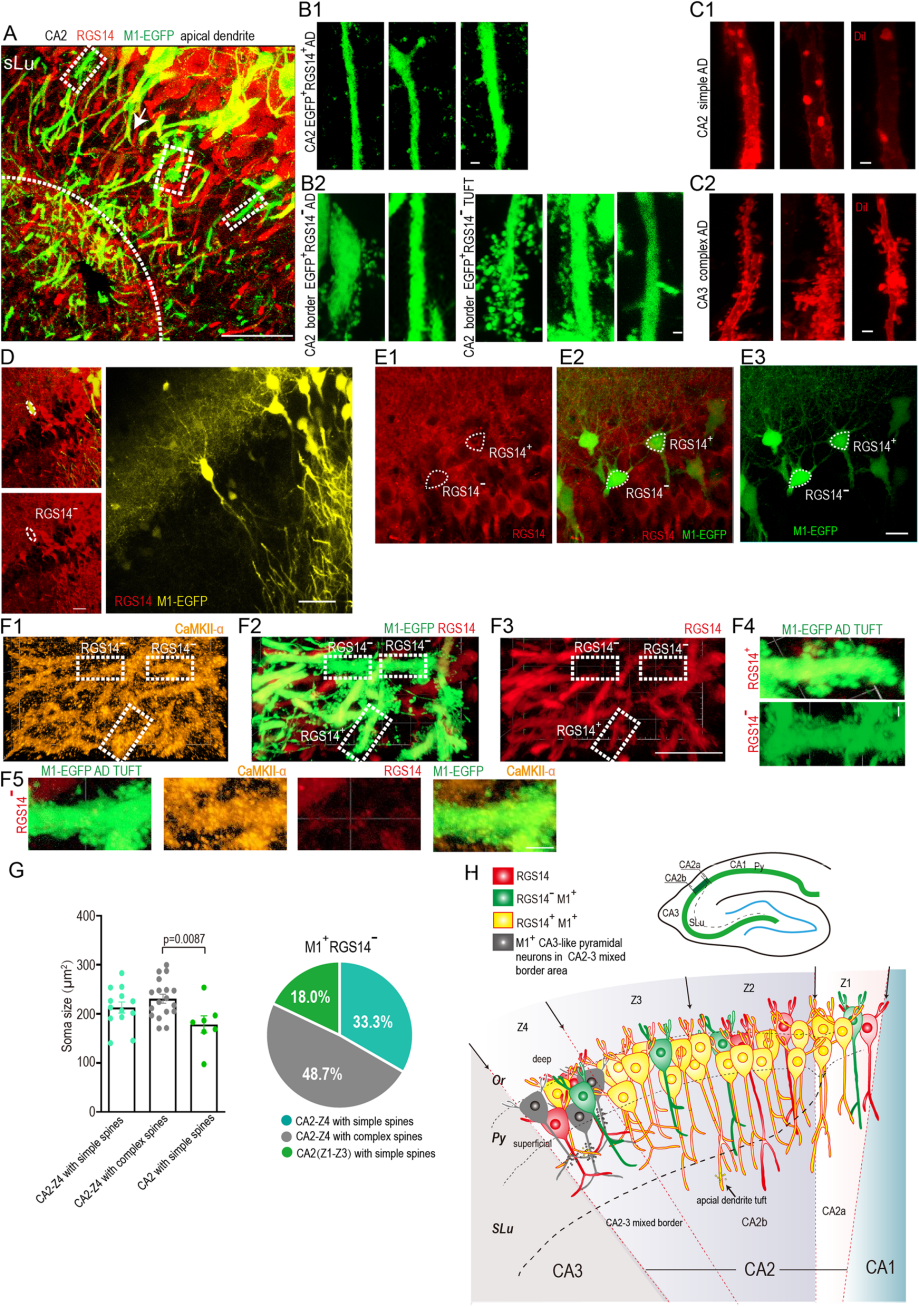
Morphology features and locations of CA2 M1^+^RGS14^−^ neurons. **A** Representative image of apical dendrites (AD) in CA2 stratum lucidumis (SLu) infected with AAV9/M1-EGFP (n = 5 mice). Dashed rectangular boxes show RGS14^−^EGFP^+^ AD of complex spines. Arrow indicates simple AD on CA2 RGS14^+^ neurons like previous research. **B1** magnified images of simple spines in AD of most M1^+^RGS14^+^ neurons in SLu layer from area as indicated by the white arrow in **A**. **B2** magnified images of complex and simple spines in main apical dendrites (left) and apical dendrites tuft (right) of some M1^+^RGS14^−^ neurons in SLu layer from dashed rectangular enclosed regions of **A**. **C1, C2** Dil stained typical CA2 simple spines and CA3 complex spines. **D** Representative images of a M1^+^RGS14^−^ neuron in AAV9/M1-EGFP infected CA2 region. **E1–E3** Representative images of a group of pyramidal neurons displaying similar triangle cell bodies but can be divided into M1^+^RGS14^+^ and M1^+^RGS14^−^ neurons in CA2 area close to CA3. **F1–F5** Z-projected IHC images displaying thorny excrescences of RGS14^+^ neurons apical dendrites tuft in SLu. Dashed rectangular boxes show M1^**+**^RGS14^+^ and M1^+^RGS14^−^ neurons co-stained with CaMKII-α (**F1**). Images shows RGS14^+^ or RGS14^−^ M1-EGFP complex apical dendrites tuft (**F4**) **G** Scatter chart (left) displaying soma size of M1^+^RGS14^−^ in CA2-Z4 and CA2. Pie chart (right) displaying the proportions of different located neurons with complex or simple spines in total M1^+^RGS14^−^ (n = 5 mice). **H** Diagram summarizing the features and locations of M1^+^RGS14^−^ neurons in CA2 Py layer. M1^+^RGS14^−^ neurons with simple spine can be detected in the CA2b region (Fig. 3B1–B4, 3E1 and H are the good examples). M1^+^RGS14^−^ neurons can be detected in both the deep and superficial layers of the CA2b Py region (Fig. 3B1), and its cell morphology will be more like the isosceles triangle. It shows neurons with complex spine in CA2-CA3 mixed border (black) supposed be CA3-like neurons. Scale bars: 2 μm (**B1, B2, C1, C2, F4**), 50 μm (**A, D**), 20 μm (**E1–E3, F1–F3, F5**)

**Fig. 5:**
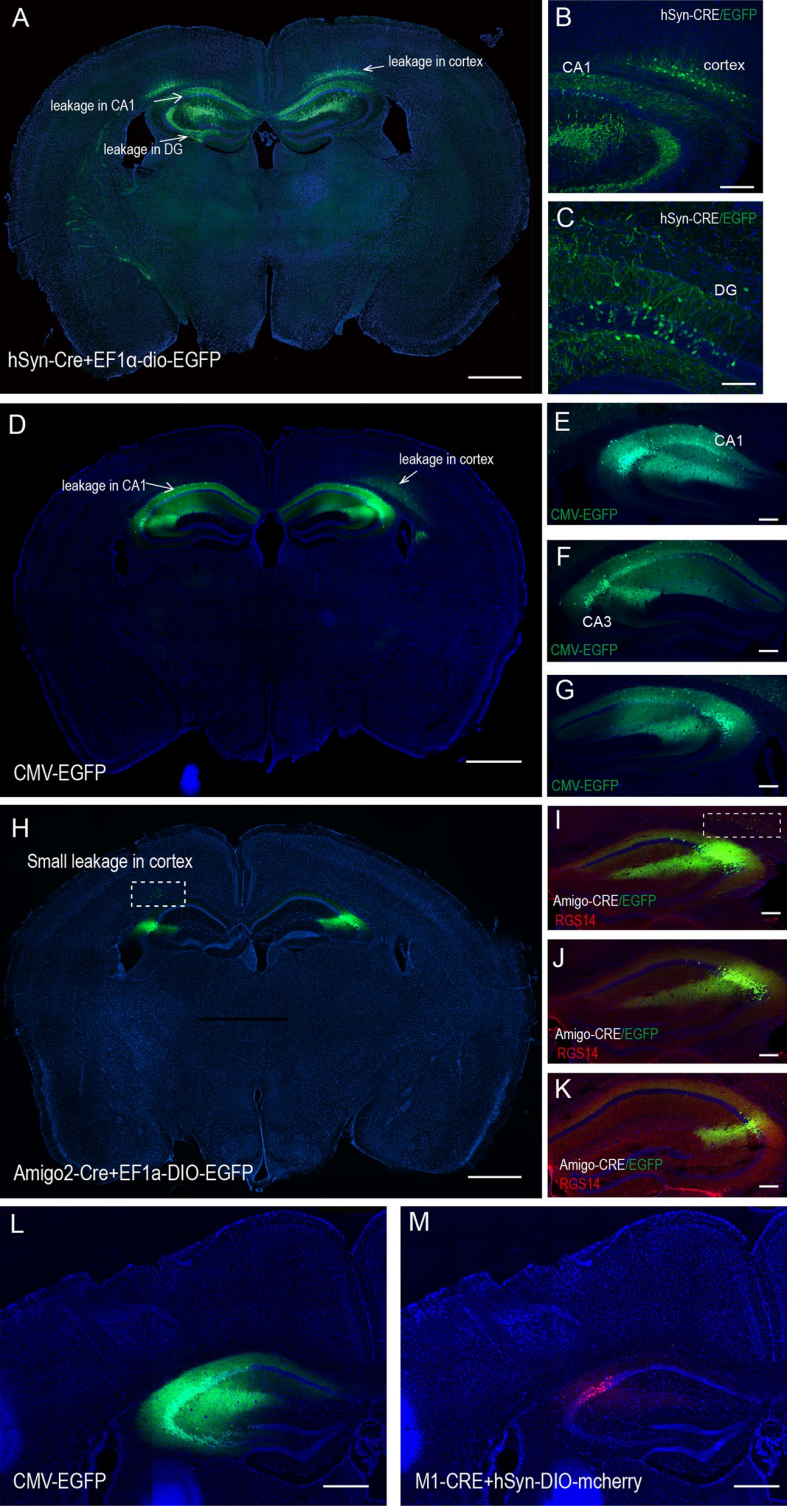
Co-injection of AAV9/M1-Cre & AAV9/hSyn-DIO-mcherry in WT dCA2 leads to the optimal expression accuracy comparing to the co-injections of hSyn-Cre&EF1α-DIO-EGFP in the WT dCA2, the single injections of AAV9/CMV-EGFP in the WT dCA2 and the single injections of AAV9/DIO-EGFP in the Amigo2-Cre dCA2. **A** image of whole brain coronal section with the co-injection of AAV9/hSyn-Cre & AAV9/EF1α-DIO-EGFP (green) in dCA2. **B** Confocal fluorescence images showing the expression of EGFP induced by hSyn-Cre in hippocampus. There were 4 forms: 1. it leaked in the sixth layer of cortex; 2. although there was fluorescence at the CA2 position, there was no representative soma of pyramidal cell; 3. there were also projections of contralateral CA3; 4. infection of hSyn-EGFP in CA1 showed the hSyn-Cre could not specifically and accurately label CA2. **C** Magnified image showing hSyn-EGFP infection in DG. **D** Image of whole brain coronal section with the infection of CMV-EGFP in dCA2. **E–G** Serial sections of WT dCA2 injected with AAV9/CMV-EGFP (n = 5 mice) showing CMV-EGFP was also expressed in CA1 and CA3 in addition to CA2. **G **Upper right corner showing CMV-EGFP expressed in the sixth cortex.** H** Whole brain fluorescence images of Amigo2-Cre transgenic mice injected with AAV9/EF1α-DIO-EGFP (n = 8 mice). **I–K** Serial sections of Amigo2-cre dCA2 expressing EGFP (green) and RGS14 (red). Amigo2-EGFP nonspecific leakage on **(H)** left corner of image and **(I)** right corner, however the Amigo2-cre transgenic mice had the accuracy and specificity of CA2 pyramidal neurons. **L** Coronal section fluorescence image of WT mice stereotaxically injected with CMV-EGFP (green). **M** Coronal section fluorescence image of WT mice stereotaxically injected with AAV9/M1-Cre & AAV9/hSyn-DIO-mcherry (red) (n = 6 mice). Scale bars: 100 μm (**C**), 200 μm (**B, E–G, I-K**), 1 mm (**A, D, H**), 500 μm (**L, M**)

**Fig. 6:**
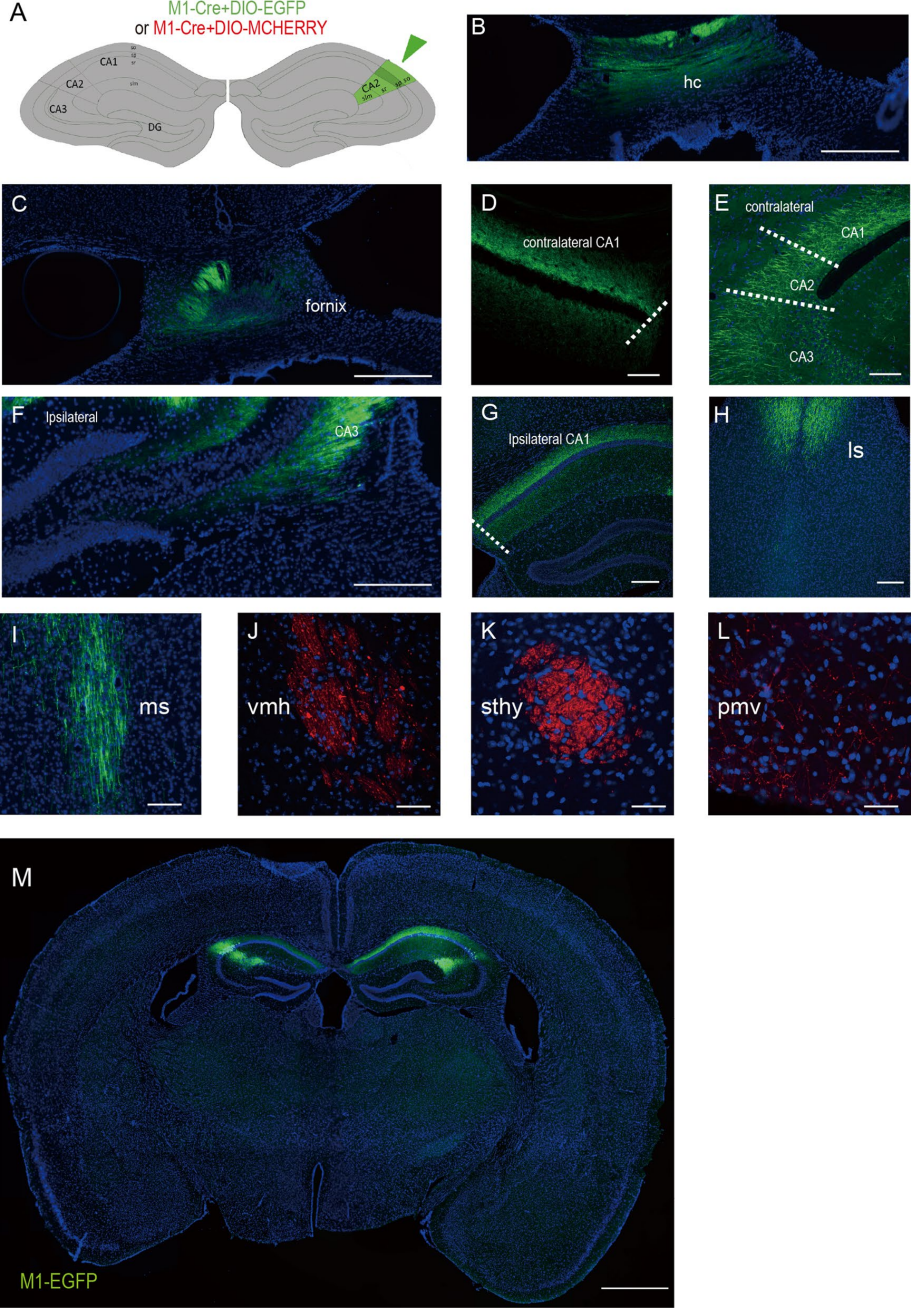
Efferent contralateral and longitudinal projections of dCA2 infected with AAV9/M1-Cre. **A** Schematic of unilateral stereotactic injection at dCA2 of WT mouse with AAV9/M1-Cre & AAV9/EF1α-DIO-EGFP (n = 10 mice). **B** Image showing M1-EGFP axons traversing to the hippocampal commissure.** C** Image showing M1-EGFP axons traversing to the hippocampal fornix. **D, E** Fluorescence images showing dCA2 unilaterally injected with M1-EGFP displayed projections to the contralateral dorsolateral CA1, CA2 and CA3 (**E**). **F, G** Images showing M1-EGFP axons had projections to ipsilateral CA3 and CA1. **H, I** Confocal images showing dCA2 bilaterally injected with M1-EGFP displayed efferent projections to septal areas LS **(H)** and MS **(I)** (n = 16 mice). **J–L** Confocal images showing dCA2 bilaterally co-injected with AAV9/M1-Cre & AAV9/hSyn-DIO-mcherry displayed longitudinal projections to vmh (**I**), sthy (**J**) and pmv (**K**) (n = 9 mice). **M** Coronal section of mouse brain infected with M1-Cre & EF1α-DIO-EGFP (M1-EGFP) in dCA2 (n = 39 mice). Stained with DAPI (blue). Scale bars: 1 mm (**M**), 500 μm (**B, C, F**), 200 μm (**G, H**), 100 μm (**D, E, I**), 50 μm (**J–L**)

**Fig. 7:**
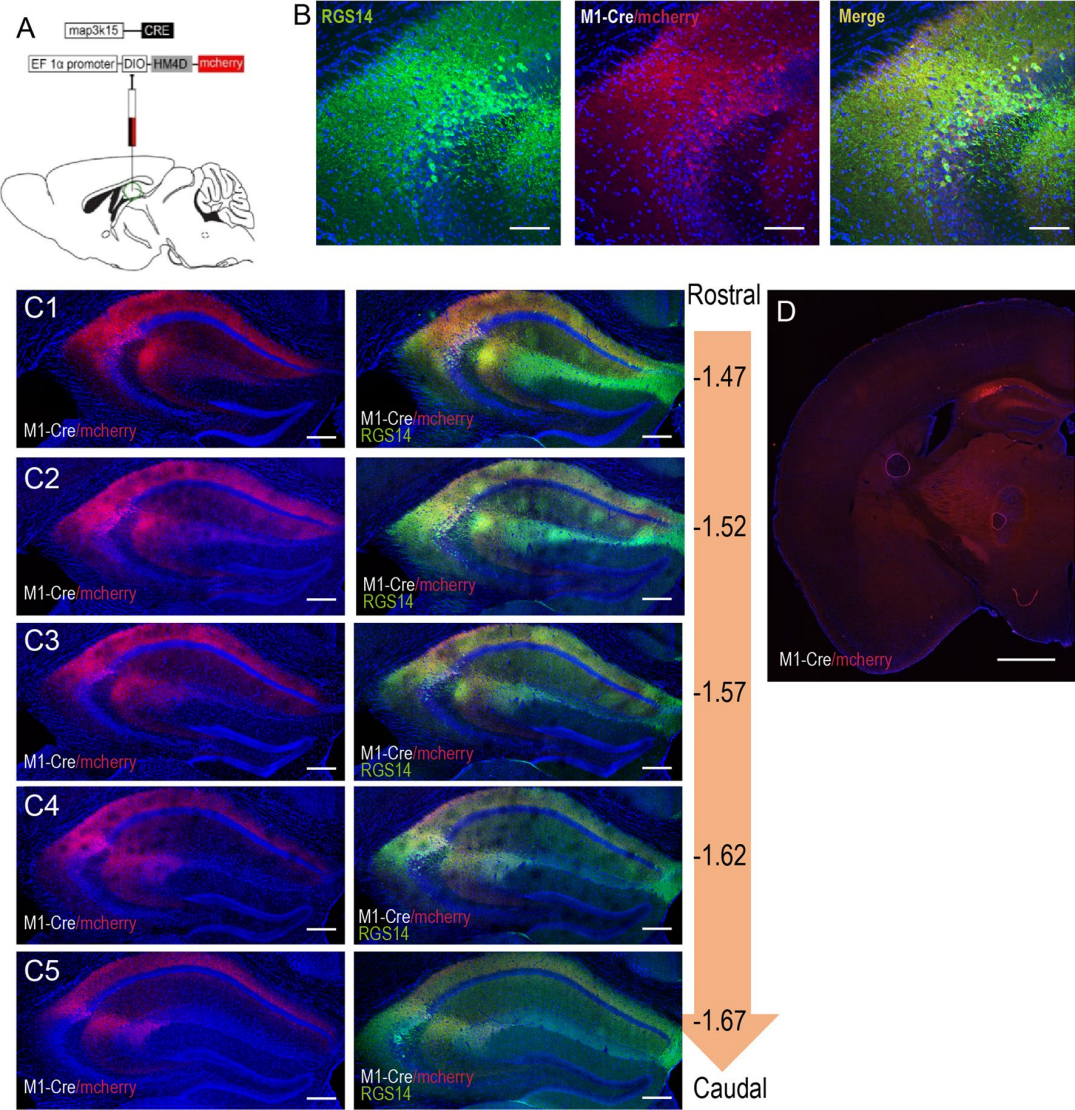
Expression pattern of the chemogenetic inhibitor DIO-hM4Di induced by M1-Cre in dCA2. **A** Schematics of stereotactic injection of AAV9/M1-Cre & AAV9/EF1α-DIO-hM4Di-mcherry in WT dCA2. **B** Magnified fluorescence images of RGS14(green), M1-Cre/DIO-hM4Di-mcherry(red) and trio-channels displaying hM4Di expressed within RGS14 stained position. **C1–C5** Images showing representative coronal serial sections of dCA2 infected with hM4Di-mcherry (red) from beak to tail (left). Images displaying mcherry located in RGS14 region (right) (n = 21 mice). **D** the fluorescence image of half-brain slice which CA2 infected with M1-Cre/DIO-hM4Di-mcherry. Scale bars: 1 mm (**D**), 200 μm (**C1–C5**), 100 μm (**B**)

**Fig. 8:**
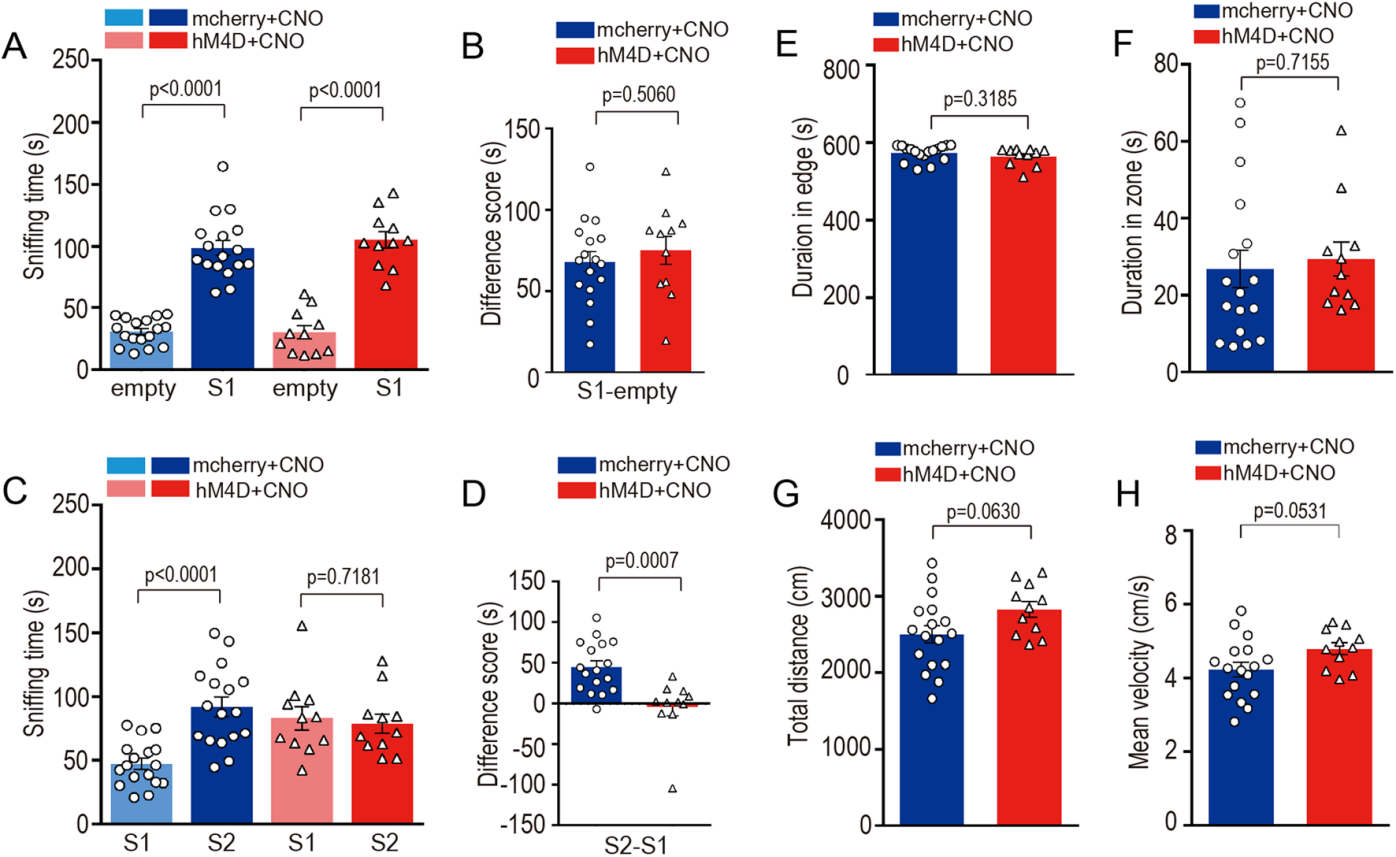
M1-Cre induced DIO-hM4Di mediated chemogenetic inhibition of dCA2 impaired social memory. **A** Sociability test: inhibition mice (red, pink) (n = 11 mice) and control mice (blue, azure) (n = 17 mice) both preferred con-specifics mouse (S1) compared to sniffing time of empty container. **B** Sniffing time difference scores (S1 minus empty) were similar (*p* = 0.5060, two-tailed Student’s *t*-test). **C** Social novelty test: only control mice (blue, azure) preferred new unfamiliar mice (S2) over S1. **D** Sniffing time difference score (S2 minus S1) of CA2-inhibited group was significantly lower than that of control group (*p* < 0.0001). **E** Open-field behavioral test: duration in edge zone of inhibition mice (dark red) and control mice (dark blue) were similar (*p* = 0.3185). **F** Open-field behavioral test: duration in central zone of inhibition mice and control mice were similar (*p* = 0.7155). **G** Open-field behavioral test: total distances of inhibition mice (dark red) and control mice (dark blue) were similar (*p* = 0.0630). **H** Open-field behavioral test: mean velocities of inhibition mice and control mice were similar (*p* = 0.0531)

For RGS14, Calretintin, Calbindin, GAD67, Parvalbumin, NeuN, mcherry, CaMKII-α, PCP4 and GFP staining, the brain sections were incubated with the primary antibodies (mouse antibody against RGS14, NeuroMab, Cat:75-170; rabbit antibody against Calretintin, Proteintech, Cat:66496-1-Ig; mouse antibody against Calbindin Proteintech, Cat:14479-1-AP; mouse antibody against GAD67, Millipore, Cat:MAB5406; mouse antibody against Parvalbumin, sigma, Cat:048M4753V; mouse antibody against NeuN, Millipore, Cat:MAB377; rabbit antibody against mcherry, Invitrogen, AB-213511 rabbit antibody against PCP4, SIGMA, HPA005792; rabbit antibody against CaMKII-α, MERCK, C6974 and rabbit antibody against GFP, Torrey Pines Biolabs, Cat:TP401) and secondary antibodies (Alexa Fluor 488 donkey anti-rabbit IgG, Invitrogen, A21206; Alexa Fluor 488 donkey anti-mouse IgG, Invitrogen, A21202 and Alexa Fluor 555 goat anti-rabbit IgG, Invitrogen, A21429). For CA2 spine imaging, RGS14 and CaMKII-α were costained on 1.5%PFA fixed brain slices with the thickness of 150 μm prepared by a vibratome. Sections were incubated with 5% FBS in 0.1% PBT for 1 h, then incubated with the primary antibodies against RGS14 (1:500) and CaMKII-α (1:500) in 3% FBS (0.05% PBT) overnight, and visualized using secondary antibody Alexa 555 (1:200) and Alexa 647 (1:200). The cells were counted on the complete coronal hippocampus images which were acquired through stitching the 10 × confocal microscopic diagrams together. RGS14^+^ cell number in RGS14 stained region was defined as total CA2 cells (Cell sum) and is normalized as 100%. The formulas for Fig. 2G are shown as below:

$${\mathrm{Proportion}}_{{\mathrm{RGS}14}^{+}{\mathrm{EGFP}}^{-}}=\frac{{\mathrm{number}}_{{\mathrm{RGS}14}^{+}}-{\mathrm{number}}_{{\mathrm{RGS}14}^{+}{\mathrm{EGFP}}^{+}}}{\mathrm{Cell \,sum}}$$

$${\mathrm{Proportion}}_{{\mathrm{RGS}14}^{+}{\mathrm{EGFP}}^{+}}=\frac{{\mathrm{number}}_{{\mathrm{RGS}14}^{+}{\mathrm{EGFP}}^{+}}}{\mathrm{Cell \,sum}}$$

$${\mathrm{Proportion}}_{{\mathrm{EGFP}}^{+}RGS14^{-}\mathrm{in \,CA}2 \,{\mathrm{}}{}}=\frac{{\mathrm{number}}_{{\mathrm{EGFP}}^{+}\mathrm{in \,CA}2}\_{\mathrm{number}}_{{\mathrm{EGFP}}^{+}{\mathrm{RGS}14}^{+}}}{\mathrm{Cell \,sum}}$$

$${\mathrm{Proportion}}_{{\mathrm{EGFP}}^{+}RGS14^{-}\mathrm{ outside \,CA}2\, {\mathrm{}}^{}}=\frac{{\mathrm{number}}_{{\mathrm{EGFP}}^{+}\mathrm{outside \,CA}2}}{\mathrm{Cell \,sum}}$$

ImageJ software (National Institutes of Health, Bethesda, USA) and ZEN (Zeiss, Oberkochen, Germany) were used to count cells and measure soma size.

### Diolistic labeling

Mice were perfused with 1.5% PFA, and the brains were postfixed by the same solution overnight. Then, 150 µm-thick sections were prepared by a vibratome and labeled by Dil-labeled Au particles using Gene gun. After 24 h at room temperature proceed with incubation with antibody to RGS14 (1:500) in 10% FBS for 16 h, and visualized using secondary antibody to mouse Alexa 647.

### Data analysis

Ordinary one-way ANOVA, post-hoc test Bonferroni’s test, and two-tailed unpaired Student’s test was used to assess statistical significance as described in the figure legends. GraphPad Prism 7 (GraphPad, CA, USA) was used for data analysis. Photoshop CS6 (Adobe, CA, USA) was used to merge confocal images to fully present the hippocampus. All the statistical data in the graphs are presented as the mean ± SEM. p < 0.05 was considered significant. All p values were shown in the figures and n represented the number of animals.

## Results

### M1 promoter displays the highest transcription activation efficiency and AAV9/M1-Cre displays the best restriction and accuracy targeting to CA2 comparing to AAV9/M2-Cre or AAV9/RGS14-Cre

Previous studies reported several CA2 markers such as PCP4, RGS14, Amigo2 and Map3k15 [13, 18, 19]. After scrutinizing the expression pattern of these markers in hippocampus in the literatures and the Allen Brain Atlas database (https://portal.brain-map.org), we decided to choose comparatively more restricted CA2 markers, RGS14 and Map3k15, for further promoter activity and specificity test. We wonder which gene and which promoter region are the best choice for CA2 highly preferential viral plasmid construction. Therefore, several vectors containing different promoter fragments from *rgs14* and *map3k15* with various lengths were constructed and tested. Using the luciferase assays for the promoter activity tests, we screened out a fragment from *map3k15* with the length of around 500 bp, which displays the highest luciferase activity (Fig. 1A). To primarily test if it also has a good specificity, the viral vectors containing different *rgs14* and *map3k15* promoters fused with a Cre gene (Fig. 1B) were packaged and injected into the dorsal CA2 region with AAV9/hSyn-DIO-mcherry. The AAV9/CMV-EGFP was co-injected with the same dosage as a control. This preliminary study indicated the best CA2 marking effects of AAV9/Map3k15 (0.5 kb)-Cre (M1-Cre) instead of AAV9/Map3k15 (2.3 kb)-Cre (M2-Cre) or AAV9/RGS14-Cre (Fig. 1C–E). Meanwhile, the co-injection results of CA2 highly preferential AAV9/M1-Cre & EF1α-DIO-mcherry system and nonspecific AAV9/CMV-EGFP virus into CA2 showed in Fig. 1C–E suggests that EGFP driven by the CMV promoter is expressed much more pervasive in hippocampus. Although the precise stereotaxic was used, it was still impossible to restrict protein expression in the CA2 region without the CA2 specific or highly preferential promoter. The specificity of M2-Cre in CA2 was slightly more accurate than that of the RGS14 promoter, but they were not as accurate as the M1-Cre.

### AAV9/M1-Cre displays a better restriction and accuracy targeting to CA2 than nonspecific AAV9/Synapsin-Cre or AAV9/CMV-EGFP and labels a group of M1^+^ RGS14^−^ neurons

To further test if our M1-cre can truly target CA2 region, serial sections were collected and thoroughly investigated 3–4 weeks after the stereotaxic coinjections of AAV9/M1-Cre & AAV9/EF1α-DIO-EGFP (or EF1α-DIO-mcherry, AAV5/EF1α-DIO-EGFP) mixture performed as before in dorsal CA2 (Fig. 2A). And RGS14 was used as a CA2 marker to delineate this region. Our results displayed that GFP was efficiently and accurately expressed through dorsal CA2 without leakage by M1 driven Cre expression (Fig. 2B1–B5). Again, the non-CA2-specific promoter hSyn or CMV driven Cre was used as a control. The same dosage of AAV9/hSyn-Cre & EF1α-DIO-EGFP co-injection led to a broader nonspecific expression beyond CA2 region. The expression of non-specific EGFP could be pervasively detected in CA1, CA3 and DG (Fig. 2C1–C5, D1–D5). Contrarily, RGS14 could be much better colocalized with M1-driven EGFP expressed cells (the percentage of EGFP^+^RGS14^+^ cell is ~ 48% of total RGS14^+^ cells) in CA2 (Fig. 2G) but much less overlapped with CMV (the percentage of EGFP^+^RGS14^+^ cell is ~ 13% of the total RGS14^+^ cells) or hSyn driven EGFP expressed cells (the percentage of EGFP^+^RGS14^+^ cell is ~ 3% of the total RGS14^+^ cells) (Fig. 2G). In conclusion, there are much less viral expressing cells outside of CA2 regions when applying M1 instead of nonspecific promoters to drive GFP expression (Fig. 2G). And over 95% of these M1-driven EGFP expressed cells are not colocalized with the interneuron markers (Fig. 2F1–F4, I) which means most M1-driven EGFP expressed cells are pyramidal neurons. Moreover, 84.7% of M1-driven EGFP pyramidal neurons in CA2 region receive the projections from Calbindin positive (CB^+^) mossy fibers from dorsal dentate gyrus (Fig. 2F5, J) which was consistent with the phenomenon observed in PCP4^+^ pyramidal neurons in dorsal CA2 [18]. One thing surprising for our immunofluorescence results is that a considerable number of M1 expressing cells does not colocalize with RGS14 (Fig. 2E1–E4). The ratio of EGFP^+^RGS14^−^ cells within RGS14 stained region (~ 16% of the total RGS14^+^ cells) for M1-Cre treatment (Fig. 2G) to EGFP^+^RGS14^+^ cells within the same region (~ 48% of the total RGS14^+^ cells) (Fig. 2G) is around 1:3, which means around 25% of M1 expressing cells does not express RGS14. This is different with the reported result showing almost 100% coexpression of STEP, PCP4 and RGS14 within CA2 region [18], and the result in another previous study showing ~ 97% overlapping between RGS14 and Amigo2 positive cells in this region [13].

### M1^+^ RGS14^−^ neurons in CA2 Py layer are typical pyramidal neurons with varied cell body morphology and CA3-like pyramidal neurons in CA2-CA3 border

To determine if those M1^+^RGS14^−^ cells are real CA2 cells instead of pyramidal neurons from CA1 or CA3, we first thoroughly re-compare the size of cell bodies in CA1, CA3 and CA2 regions as delineated in Fig. 3A1–A3. There are some of them are in the CA2 Z3-Z4 border or in the CA2 Z2-Z3 area according to previous definition of CA2 (Fig. 3B1–B3) [12]. We found the average size of cell bodies in CA2 regions is obviously larger than CA1 pyramidal neurons (Additional file 1: Fig. S1A). And M1^+^RGS14^−^ neurons from CA2 pyramidal cell (Py) layer also show the larger cell body size than CA1 pyramidal neurons in Py layers as previously reported [20]. M1^+^RGS14^−^ neurons from stratum oriens (Or) layer are also quantified and their body size is close to the body size of PV and CB interneurons but not CR interneurons in Py layer (Additional file 1: Fig. S1A). To further investigate the features of these M1^+^RGS14^−^ cells, another CA2 marker PCP4 was utilized and indicated the high colocalization with RGS14 staining cells (Fig. 3C1–C4, D1–D4). PCP4/M1 and RGS14/M1 have similar non-colocalization ratio in the whole CA2 (Additional file 1: Fig. S1B, C) or in different CA2 layers (Additional file 1: Fig. S1D). Next, we used CaMKII-α to label those M1^+^RGS14^−^ cells in CA2-Z3 or CA2-Z4 regions (Fig. 3B4, E1–E5) and it does demonstrate that they are CaMKII^+^. Sometimes even a large area of CA2 cells can be detected as M1^+^RGS14^−^ CaMKII^+^ cells (Fig. 3F1–F4). Those pieces of evidence highly suggest most of M1^+^RGS14^−^ neurons are pyramidal neurons.

Meanwhile, we carefully observed dendritic spines especially spines in apical dendrites of both M1^+^RGS14^−^ and M1^+^RGS14^+^ neurons in stratum lucidum (SLu) layer of CA2 (Fig. 4A, B1–B2). We found most of M1^+^RGS14^+^ cells have typical CA2 simple spines as shown in Fig. 4B1 and C1, and comparatively much rare complex spines can be detected in this group of cells. However, a different case was found in M1^+^RGS14^−^ cells. Besides pyramidal neurons with simple spines were detected in this group of cells, the neurons with complex spines were also detected especially in CA2-CA3 mixed borders (Fig. 4B2) which used to be more commonly observed in CA3 pyramidal neurons (Fig. 4C2). Since CA2–CA3 has a mixed border, the M1^+^RGS14^−^ neurons with complex spines in this region are possibly the CA3 neurons. Meanwhile they are in RGS14 covered region and stained with CaMKII (Fig. 3F1–F4), we call them CA3-like pyramidal neurons. We quantified the soma size and percentage of neurons containing complex spine and neurons with simple spines in CA2–CA3 border. We found the soma size of M1^+^RGS14^−^ with complex spines in these region is bigger than that of the M1^+^RGS14^−^ neurons with simple spines (Fig. 4G, the bar graph), which does mean M1^+^RGS14^−^ with complex spines in this region are similar with CA3 neurons. And 48.7% of M1^+^RGS14^−^ neurons belong to this situation. Around 51% of M1^+^RGS14^−^ neurons have simple spines and are still normal CA2 pyramidal neurons (Fig. 4G, the pie chart), which are stained with CaMKII (Fig. 3B4). Last, we closely study the morphology of M1^+^RGS14^−^ and M1^+^RGS14^+^ cell bodies. Surprisingly both of them appear diverse shapes from typical pyramidal form (Fig. 4D) to other kind of shapes such as the triangle ones as shown in Fig. 4E1–E3. The complex spines were also observed on the apical dendritic tuft in both of RGS14^+^ and RGS14^−^ spines in SLu layer as shown in Fig. 4F1–F5.

We summarize what we found about CA2 M1^+^ neurons in Fig. 4H, most of M1^+^ cells can colocalized with RGS14 as M1^+^RGS14^+^ neurons (yellow). But some of them are M1^+^RGS14^−^ neurons. They can be detected in different area of CA2. Half of them are normal CA2 pyramidal neurons. But half of them are CA3-like neurons (grey) within CA2–CA3 mixed border (Z4 area) or CA2b region tightly close to the border (Z4–Z3 border). The case to detect CA1-like M1^+^RGS14^−^ neurons in CA1–CA2 border is very rare comparing to the possibility to detect CA3-like M1^+^RGS14^−^ neurons in CA2–CA3 border.

### Combined administration of AAV9/M1-Cre & AAV9/EF1α-DIO-mcherry achieves an even less leakage and the same accuracy targeting to dorsal CA2 as the single dosage of AAV9/EF1α-DIO-mcherry to Amigo2-Cre mice

The mixed usage of two viruses usually has a lower site accuracy than the single viral usage. It is consistent with our results in Fig. 5. Compared to a broad diffusion to the whole hippocampus and even the cortex caused by mixed viral usage of AAV9/hSyn-Cre & AAV9/EF1α-DIO-EGFP in the dorsal CA2 (dCA2) region (Fig. 5A–C), the single usage of AAV9/CMV-EGFP has a lower diffusion beyond hippocampus (Fig. 5D–G). But both of these two applications cannot restrict the injected viruses just within dCA2. The application of AAV9/EF1α-DIO-EGFP into CA2 specific Amigo2-Cre mice efficiently help to increase the specificity of EGFP expression in dCA2, while a comparatively low level of leakage into cortex still can be detected (Fig. 5H–K). Surprisingly, our combined administration of AAV9/M1-Cre & AAV9/EF1α-DIO-mcherry achieved the optimal restriction of fluorescent protein in dCA2 region and lowest viral leakage to the peripheral regions (Fig. 5M, Additional file 1: Fig. S2A1-A3, B1-B4, C1-C4) comparing to CMV (Fig. 5L) or even Amingo2 driven EGFP expression (Fig. 5I–K). The small leakage as shown in Additional file 1: Fig. S2 A1 actually used to be mistakenly confused with the neurons in CA2a area in many other reports. The small leakage as shown in the Additional file 1: Fig. S2 A2 was confirmed to mostly take place in the Or layer. And the small leakage in CA3 as shown in Additional file 1: Fig. S2 A3 is very rare.

### AAV9/M1-Cre can be efficiently utilized for anterograde neural tracing and neural activity manipulation in dCA2

To test if M1 driven system can be utilized for anterograde neural tracing, AAV9/M1-Cre & AAV9/EF1α-DIO-mcherry or AAV9/M1-Cre & AAV9/EF1α-DIO-EGFP were injected and fluorescent signals were traced (Fig. 6A). We successfully observed projections from CA2 to the following regions: hippocampal commissure and fornix (Fig. 6B, C), contralateral CA1, CA2 and CA3 regions (Fig. 6D, E), ipsilateral CA1 and CA3 (Fig. 6F, G), lateral septum (LS), medial septum (MS), ventromedial hypothalamus (VMH), striohypothalamic nucleus (STHY) and ventral premammillary nucleus (PMV) (Fig. 6I–L). Meanwhile, all of the serial sections for neural tracing were carefully examined to guarantee that there is no obviously discernable leakage as in Figs. 5M and 6M. In our tracing experiments, some known CA2 downstream regions can be constantly recapitulated [16, 21, 22]. But VMH, STHY and PMV are the new projected regions we detected using this M1 system. What we find for CA2 projection tracing further prove that our system can accurately label CA2 and be applicable for CA2 specific neural tracing.

Last we wonder if AAV9/M1-Cre virus suitable to be applied into neural activity regulation through chemogenetic manipulation in CA2 region. To test chemogenetic efficiency, AAV9/M1-Cre & AAV9/EF1α-DIO-hM4Di-mcherry were co-injected into CA2 (Fig. 7A) and successfully expressed after 4 weeks of injection (Fig. 7B–D). Then the neural activity in CA2 was inhibited by CNO induction in the viral injected mice. The mice with CA2 neural inhibition displayed the classical social memory defect in three-chamber social test (Fig. 8A–D) without the defects of social ability, anxiety level or motion ability (Fig. 8E–H).

## Discussion

Overall, we successfully developed a CA2 highly preferential Cre viral system to label and manipulate neural activity in CA2 region. It can efficiently drive CA2 pyramidal neurons as what Amigo2-Cre transgenic mice does. Meanwhile, it even displayed an extraordinary CA2 targeting accuracy comparing to most of current available targeting systems for CA2 and it is much more flexible to be utilized into different mice to realize the CA2 specific manipulation and gene deletion.

Moreover, our mini-Map3k15 driving Cre system enriched pyramidal neuron types able to be manipulated in CA2. Using this system, we highly preferentially labeled CA2 neuronal cells containing both M1^+^RGS14^+^ and M1^+^RGS14^−^ pyramidal neurons in Py layers of CA2. M1^+^RGS14^+^ neurons occupy around 80% of total M1^+^ cells and they are all typical CA2 pyramidal neurons. For those M1^+^RGS14^−^ neurons in Py layers of CA2, their cell body size and CaMKII presence still announce their typical CA2 pyramidal neuron identity. Further studies on spine morphology indicate that the M1^+^RGS14^−^ CA2 like pyramidal neurons in CA2-CA3 border actually are the mixtures of normal CA2 neurons (with simple spines) and CA3-like neurons (with complex spines). Half of M1^+^RGS14^−^ neurons are the normal CA2 pyramidal neurons, which occupy around 10% of total M1^+^ neurons in CA2. Some of M1^+^RGS14^−^ cells (around 2–3% of total M1^+^ cells) does localize in Or or SLu layer of CA2. They are probably GAD67^+^ interneurons with other novel markers need to be further explored. Meanwhile CA2 neurons displayed diverse morphology of pyramidal neuron cell bodies not only in M1^+^RGS14^−^ neurons in CA2 Py layers, but also in M1^+^RGS14^+^ or regular CA2 neurons in Py layer sometimes. This finding transform our former impression on pyramidal neurons (more homogenic in morphology and function comparing to the interneurons) in hippocampus. But it is consistent with the previous reports that CA2 does have multiple pyramidal neuron types [12, 23].

One more thing needs to be noticed. Given the 97% overlapping between RGS14 with Amigo2 as previously reported, ~ 10% of total M1^+^ neurons in CA2 Py layers cannot colocalize with Amigo2. That is, this portion of M1^+^RGS14^−^ pyramidal neurons could not be labeled by Amigo2-Cre mice. And it won’t be surprised if this group of pyramidal neurons have different axonal projections with M1^+^RGS14^+^ or Amigo2^+^RGS14^+^ pyramidal neurons in CA2. And this also could explain why we could trace CA2 projecting axons to VMH, STHY and PMV using our M1-Cre system whereas this result could not be recapitulated in Amigo2-Cre mice. Similar phenomena were observed in other Cell-type specific Cre models [24, 25]. The issue here is that AAV-M1-Cre viral tool induced preferential GFP expression in CA2 while it also induced the GFP expression in some CA3-like pyramidal neurons in CA2-CA3 border area. Those CA3 like pyramidal neurons in the border are almost the same as CA3 neurons though they are covered by CA2 marker staining signals such as RGS14 or Amigo2. Our preferential CA2 targeting system is still hard to occlude all CA3-like neurons in CA2-CA3 border area or interneurons from its own neuron pool to get completely clean CA2 pyramidal neurons, so it is hard to make a clear conclusion which types of cells really projects to VMH, STHY or PMV. How to accurately separate CA2/CA3 cells from CA2-CA3 mixed border is still a challenge for us. Yong and Song recently presented the molecular evidence of mixed CA2 and CA3 neurons in the CA2-CA3 border using FISH assay [11]. But in order to ultimately characterize the neural types in CA2-CA3 border area, the more comprehensive transcriptomic or proteomic analysis should be performed in the future to clearly find out the molecular basis of neurons in this region. Another possible reason that VMH, STHY and PMV could not be traced out in Amigo2-Cre mice might be due to the difference between AAV serotypes or different viral titration leading to varied axonal transport efficiency as reported previously [26, 27].

To further increase the accuracy and usage of the CA2 specific viruses, the following strategies should be considered in the future. First, a more restricted AAV serotypes such as AAV2 or AAV5 is worth testing. Second, the viral tools for different purposes in CA2 region should be developed. An AAV/M1-Cre-EGFP (mcherry) for neural labeling/tracing and an AAV/M1-Cre-hM4Di (or hM3Dq) for neural activity regulation should be generated separately so that one viral usage can be applied instead of using mixed viruses. Third, it is uncertain if AAV/M1-Cre or Amigo2-Cre can accurately label ventral CA2. But the in-situ hybridization (ISH) data for Amigo2, RGS14 and Map3k15 in Allen Brain Atlas display the expression discrepancy of these genes in anterior and posterior part of the hippocampus which means they might not be able to correctly label the posterior CA2 as well as the anterior CA2. Otherwise, CA2 interneurons definitely need to be labeled and manipulated separately to study their own functions in the future. It is necessary to be mentioned that M1-Cre virus is obviously hard to label most of interneurons. GAD67/Map3k15 colocalization events observed in Fig. 2H are rare and only limited in Or layer maybe due to the low viral infection efficiency (~ 2%) to the non Py cells layer. Besides, another more plausible reason is that Map3k15 proteins are only expressed in interneurons at a very low level. This at least gives us a hint: to explore interneurons in CA2, it is worth screening some novel interneuron markers with certain CA2 features. So, it would be very intriguing to search and find new CA2 markers in order to develop new CA2 highly preferential viral tools targeting CA2 interneurons and posterior CA2 area. Though current CA2 highly preferential M1-Cre viral system is not a perfect tool for CA2 targeting, it is still a useful new tool to combine application flexibility, excellent expression efficiency and high degree of CA2 targeting accuracy. And hopefully this CA2 highly preferential M1-Cre system can be utilized to further deepen and expand the understanding of CA2 functions in the future.

## Supplementary Information

**Additional file 1: Figure S1.** for Figure 3 Quantitative analysis for soma size in different types of hippocampus neurons, and M1/PCP4 and M1/RGS14 co-staining comparisons. A Quantitative column chart displaying the soma size of cells in hippocampus. For measurements, images were examined using image J by delineating the soma fluorescence edge. The surface areas of CA1 pyramidal neurons, CA3 PN and CA2-EGFP^+^ were quantified by measuring AAV9/M1-EGFP-expressing neurons in IHC images. The surfaced areas of PV, CB and CR were quantified by measuring antibodies stained in IHC images. All means were calculated from no less than 5 brain slices of each biological sample. Data are presented as mean ± SEM. B Scatter chart displaying the proportions of M1^+^PCP4^-^ and M1^+^RGS14^-^ neurons in the total Map3k15-EGFP neurons. The proportions of PCP4-failed expressed and RGS14-failed expressed neurons in the EGFP were similar. C Pie charts displaying the proportions of M1^+^PCP4^-^ and M1^+^PCP4^+^ in Map3k15-EGFP, and the proportions of M1^+^RGS14^-^ and M1+RGS14+ in M1-EGFP. D Pie charts displaying the proportions of M1^+^PCP4^-^ and M1^+^RGS14^-^ neurons located in CA2 Or and Py. The proportions of M1^+^PCP4^- ^in Py and Or are the number of PCP4-in Py or Or divided by total PCP4-failedexpressed neurons number. **Figure S2.** for Figure 5 Description of M1^-^EGFP^+^ neurons outside CA2 region, and expression pattern of the AAV9/hSyn-DIO-mcherry and M1-CRE co-injection system. A1-A3 Images of M1-EGFP co-stained with RGS14. Box in A1 displays RGS14^-^EGFP^+^ neurons considered as the neuron outside RGS14 stained region. Dash-line boxes in A2 and A3 display virus leakage in the case of small probability. D1-D4, E1-E4 Coronal serial sections of dCA2 from two different mice infected with AAV9/hSyn-DIO-mcherry and M1-CREfrom head to tail. Scale bars: 200 μm, 500 μm.

## Data Availability

The datasets used or analyzed during the current study are available from the corresponding author on reasonable request.
