# Supplementary material for: A new AAV tool for highly preferentially targeting hippocampal CA2
Source: Mol Brain. 2023 Jun 11;16:50. doi: 10.1186/s13041-023-01038-6 (PMC10257852; doi:10.1186/s13041-023-01038-6)
Supplement: Supplementary file 1 — Additional file 1: Figure S1. for Figure 3 Quantitative analysis for soma size in different types of hippocampus neurons, and M1/PCP4 and M1/RGS14 co-staining comparisons. A Quantitative column chart displaying the soma size of cells in hippocampus. For measurements, images were examined using image J by delineating the soma fluorescence edge. The surface areas of CA1 pyramidal neurons, CA3 PN and CA2-EGFP+ were quantified by measuring AAV9/M1-EGFP-expressing neurons in IHC images. The surfaced areas of PV, CB and CR were quantified by measuring antibodies stained in IHC images. All means were calculated from no less than 5 brain slices of each biological sample. Data are presented as mean ± SEM. B Scatter chart displaying the proportions of M1+PCP4- and M1+RGS14- neurons in the total Map3k15-EGFP neurons. The proportions of PCP4-failed expressed and RGS14-failed expressed neurons in the EGFP were similar. C Pie charts displaying the proportions of M1+PCP4- and M1+PCP4+ in Map3k15-EGFP, and the proportions of M1+RGS14- and M1+RGS14+ in M1-EGFP. D Pie charts displaying the proportions of M1+PCP4- and M1+RGS14- neurons located in CA2 Or and Py. The proportions of M1+PCP4- in Py and Or are the number of PCP4-in Py or Or divided by total PCP4-failedexpressed neurons number. Figure S2. for Figure 5 Description of M1-EGFP+ neurons outside CA2 region, and expression pattern of the AAV9/hSyn-DIO-mcherry and M1-CRE co-injection system. A1-A3 Images of M1-EGFP co-stained with RGS14. Box in A1 displays RGS14-EGFP+ neurons considered as the neuron outside RGS14 stained region. Dash-line boxes in A2 and A3 display virus leakage in the case of small probability. D1-D4, E1-E4 Coronal serial sections of dCA2 from two different mice infected with AAV9/hSyn-DIO-mcherry and M1-CREfrom head to tail. Scale bars: 200 μm, 500 μm. [file 13041_2023_1038_MOESM1_ESM.docx]

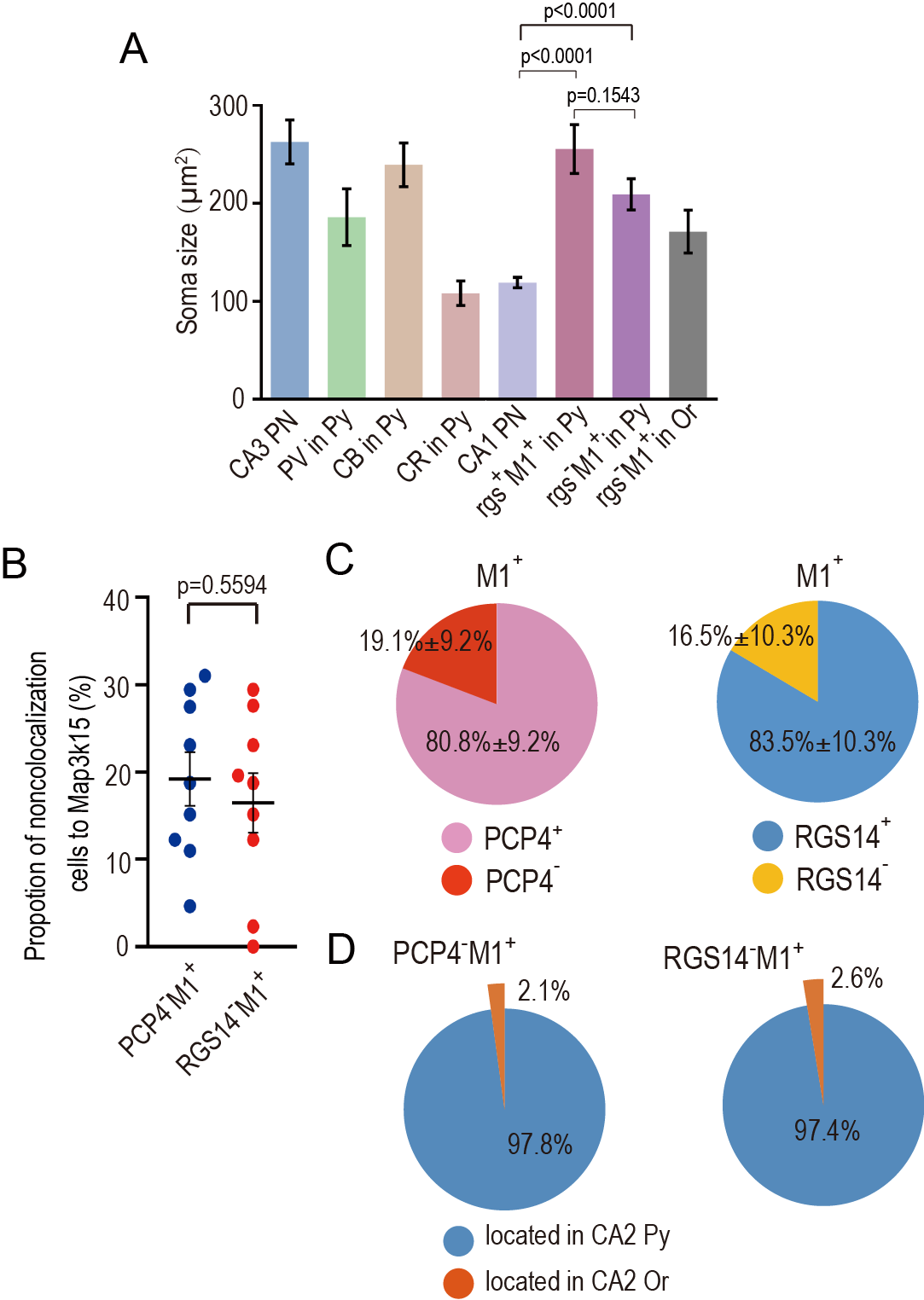


**Figure S1 for Figure 3 Quantitative analysis for soma size in different types of hippocampus neurons, and M1/PCP4 and M1/RGS14 co-staining comparisons. A** Quantitative column chart displaying the soma size of cells in hippocampus. For measurements, images were examined using image J by delineating the soma fluorescence edge. The surface areas of CA1 pyramidal neurons (PN), CA3 PN and CA2-EGFP^+^ were quantified by measuring AAV9/M1-EGFP-expressing neurons in IHC images (n=4 mice). The surfaced areas of PV, CB and CR were quantified by measuring antibodies stained in IHC images (n=3 mice). All means were calculated from no less than 5 brain slices of each biological sample. Data are presented as mean ± SEM. **B** Scatter chart displaying the proportions of M1^+^PCP4^-^ and M1^+^RGS14^-^ neurons in the total Map3k15-EGFP neurons. The proportions of PCP4-failed expressed and RGS14-failed expressed neurons in the EGFP were similar (p=0.5594) (n=4 mice). **C** Pie charts displaying the proportions of M1^+^PCP4^-^ and M1^+^PCP4^+^ in Map3k15-EGFP (red and pink), and the proportions of M1^+^RGS14^-^ and M1^+^RGS14^+^ in M1-EGFP (yellow and blue). **D** Pie charts displaying the proportions of M1^+^PCP4^-^ and M1^+^RGS14^-^ neurons located in CA2 Or and Py. The proportions of M1^+^PCP4^-^ (M1^+^RGS14^-^) in Py and Or are the number of **PCP4^-^ (RGS14^-^)** in Py or Or divided by total PCP4-failed (RGS14-failed) expressed neurons number (n=4 mice)**.**


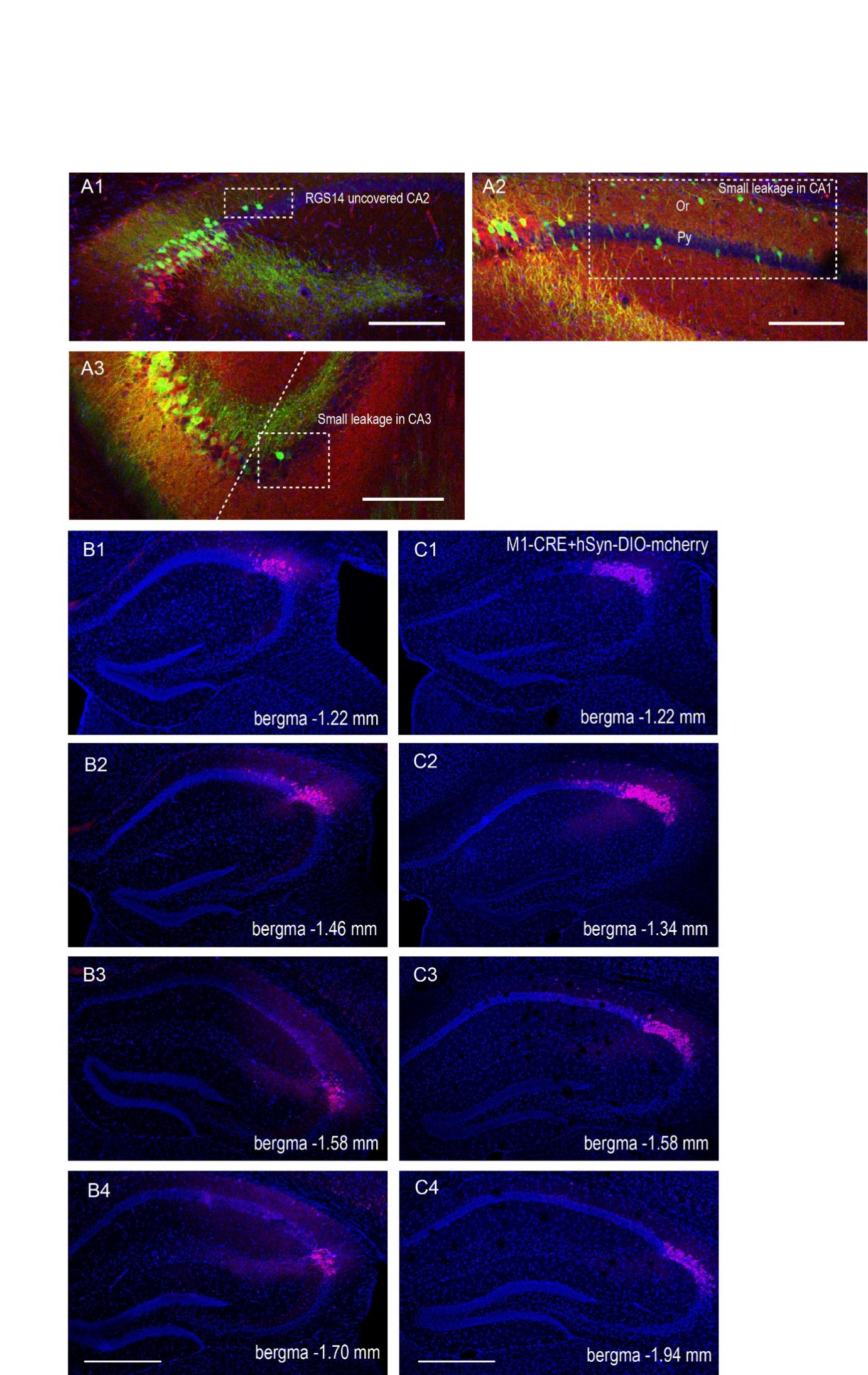


**Figure S2 for Figure 5 Description of M1-EGFP^+^ neurons outside CA2 region, and expression pattern of the AAV9/hSyn-DIO-mcherry and M1-CRE co-injection system. A1-A3** Images of M1-EGFP co-stained with RGS14. Box in **A1** displays RGS14^-^EGFP^+^ neurons considered as the neuron outside RGS14 stained region. Dash-line boxes in **A2** and **A3** display virus leakage in the case of small probability. **D1-D4, E1-E4** Coronal serial sections of dCA2 from two different mice infected with AAV9/hSyn-DIO-mcherry and M1-CRE (red) from head to tail. Scale bars: 200 μm (A1-A3), 500 μm (B1-B4 C1-C4).
